# Endocytosis: A Turnover Mechanism Controlling Ion Channel Function

**DOI:** 10.3390/cells9081833

**Published:** 2020-08-04

**Authors:** Irene Estadella, Oriol Pedrós-Gámez, Magalí Colomer-Molera, Manel Bosch, Alexander Sorkin, Antonio Felipe

**Affiliations:** 1 Molecular Physiology Laboratory, Departament de Bioquímica i Biomedicina Molecular, Institut de Biomedicina (IBUB), Universitat de Barcelona, 08028 Barcelona, Spain; irene.estadella@gmail.com (I.E.); oriolpedros@hotmail.com (O.P.-G.); magali@colomer.cat (M.C.-M.); mbosch@ccit.ub.edu (M.B.); 2 Centres Científics i Tecnològics de la Universitat de Barcelona (CCiTUB), Universitat de Barcelona, 08028 Barcelona, Spain; 3 Department of Cell Biology, School of Medicine, University of Pittsburgh, Pittsburgh, PA 15261, USA; sorkin@pitt.edu

**Keywords:** ion channels, endocytosis, turnover, ubiquitination

## Abstract

Ion channels (IChs) are transmembrane proteins that selectively drive ions across membranes. The function of IChs partially relies on their abundance and proper location in the cell, fine-tuned by the delicate balance between secretory, endocytic, and degradative pathways. The disruption of this balance is associated with several diseases, such as Liddle’s and long QT syndromes. Because of the vital role of these proteins in human health and disease, knowledge of ICh turnover is essential. Clathrin-dependent and -independent mechanisms have been the primary mechanisms identified with ICh endocytosis and degradation. Several molecular determinants recognized by the cellular internalization machinery have been discovered. Moreover, specific conditions can trigger the endocytosis of many IChs, such as the activation of certain receptors, hypokalemia, and some drugs. Ligand-dependent receptor activation primarily results in the posttranslational modification of IChs and the recruitment of important mediators, such as β-arrestins and ubiquitin ligases. However, endocytosis is not a final fate. Once internalized into endosomes, IChs are either sorted to lysosomes for degradation or recycled back to the plasma membrane. Rab proteins are crucial participants during these turnover steps. In this review, we describe the major ICh endocytic pathways, the signaling inputs triggering ICh internalization, and the key mediators of this essential cellular process.

## 1. Introduction

Ion channels (IChs) are transmembrane proteins that form pores and drive selective ions through cell membranes. IChs can be classified according to their mechanism of opening: voltage-dependent, ligand-dependent, and mechanically-dependent [1]. In addition to the sodium–potassium pump, IChs participate in the formation of an electrochemical gradient that contributes to the membrane potential. These proteins are crucial in several physiological processes, such as muscle contractions and nerve impulses, as well as several mechanisms of cellular signaling, such as cell proliferation and apoptosis and lymphocyte activation [2,3,4].

Their abundance, proper cell-surface localization, and intrinsic properties determine the activity of IChs. Thus, cells can regulate IChs activity quantitatively (the number of channels) or qualitatively (alterations to their biophysical properties) [5]. Quantitatively, gene/protein expression and association with several ancillary subunits influence the abundance of ICh membranes [6]. However, once inserted into the plasma membrane, IChs can be internalized and either recycled or degraded. Therefore, the membrane abundance of IChs relies on a balance between secretory and endosomal trafficking. The disruption of this balance is associated with diseases, especially those linked with aging and neurodegeneration [7,8,9]. Overall, the endocytic system has emerged as a crucial mechanism in the regulation of cell signaling and membrane dynamics, in addition to nutrient uptake and signal transduction initiated by cell surface stimuli, as well as the regulation of cellular metabolism and cell-to-cell communication.

The endocytic network starts with the internalization of the cargo protein via clathrin-dependent and -independent mechanisms. Internalized vesicles fuse to early endosomes (EEs), which mature into late endosomes (LEs). Early and late endosomes sort cargo destined for one of two fates: they are recycled—by transport to the plasma membrane or to a secretory pathway compartment—or degraded in the lysosomes [10,11]. Moreover, cargo proteins recycle to the cell surface by two mechanisms: (i) the fast recycling pathway, directly from the EEs, and (ii) the slow recycling pathway, using specialized recycling endosomes (REs), which are frequently clustered in the perinuclear-localized endocytic recycling compartment (ERC) [11].

In this review, we focus on the predominant endocytic pathways used by IChs, the stimuli triggering their internalization, and the essential mediators of these processes.

## 2. Endocytic Pathways

### 2.1. Clathrin-Dependent Endocytosis

Clathrin-mediated endocytosis (CME), the most studied endocytic pathway, is a common route of IChs internalization [12,13,14,15,16,17]. CME involves the recruitment of transmembrane proteins (cargo) into small areas of the plasma membrane coated with clathrin on the cytoplasmic face of the membrane (termed clathrin-coated pits, CCPs). Next, the coated membrane invaginates further until the clathrin-coated vesicle (CCV) carrying cargo is pinched off. The main component of CCPs and CCVs, clathrin, is a trimer of heavy chains (170 kDa), each associated with a light chain (25 kDa), to form a clathrin triskelion that polymerizes, forming a hexagonal coat covering the membrane [18]. Clathrin cannot bind to the lipid bilayer and requires adaptors to initiate the formation of CCPs. In fact, more than 50 ancillary cytosolic proteins are involved in CCP formation, invagination, and CCV budding. Free CCVs are rapidly uncoated and fused with early endosomes [19,20,21].

**Figure 1:**
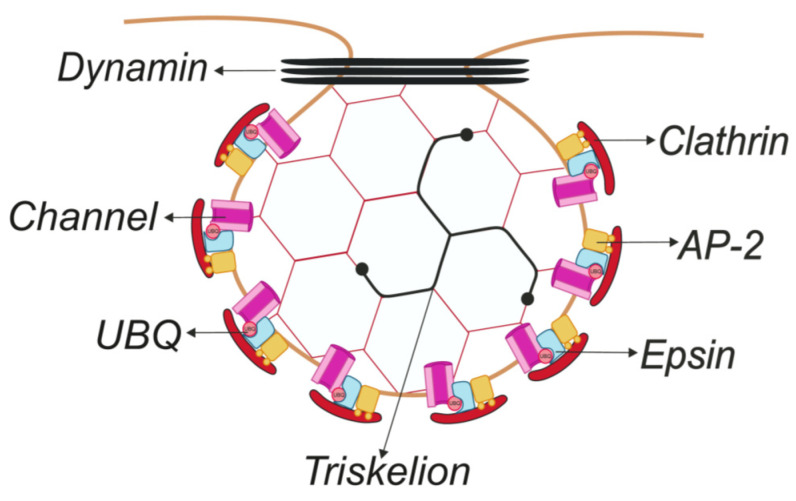
Schematic representation of the clathrin-coated pit (CCP) structure. Clathrin, the major protein in CCPs, forms a triskelion that polymerizes to form a hexagonal coat that covers the membrane. The AP-2 complex links clathrin to the membrane and coordinates the assembly of the coat with cargo proteins and lipids. In addition, epsins couple ubiquitinated membrane proteins into CCPs and contribute to the membrane curvature during the formation of clathrin-coated buds. Finally, dynamin catalyzes the constriction of the “neck” of the membrane invagination and the scission of the clathrin-coated vesicles (CCVs) from the plasma membrane. See the text for further details. Color code: red, clathrin; orange, AP-2 complex; blue, epsin; black, dynamin; light red circle, ubiquitin; and pink, ICh.

Cargo recruitment is achieved by the recognition of internalization signals (linear sequence motifs, conformational determinants, and covalent modifications) predominantly located in the cytosolic region of cargo proteins. The AP-2 complex and other adaptors, named clathrin-associated sorting proteins (CLASPs), which are located in the inner layer of clathrin coats, bind cargo [22,23]. CME also requires the action of dynamin, which catalyzes the constriction of the neck of membrane invagination, leading to the scission of a CCV (Figure 1) [19,20,21].

#### 2.1.1. Linear Sequences

Tyrosine-based motifs (YXXΦ) are involved in the CME of IChs. The voltage-dependent K^+^ channel Kv7.1 contains a C-terminal tyrosine signal (^662^YEQL^665^). The alteration of this motif prevents the AP-2- and clathrin-dependent internalization of Kv7.1 upon alpha-1-adrenergic receptor (α_1_-AR) activation [14]. The ATP-sensitive K^+^ (K_ATP_) channel, composed of Kir6.2 and the sulfonylurea receptor (SUR1) subunits in pancreatic cells, is involved in insulin secretion. K_ATP_ channels undergo rapid CME that is dependent on a tyrosine signal (^330^YSKF^333^) located in the C-terminus of Kir6.2. The disruption of this motif abolishes channel endocytosis, elevating channel surface expression and suppressing insulin secretion, thereby causing permanent neonatal diabetes mellitus [7]. The inward rectifier potassium channel Kir1.1 undergoes CME through an alternative tyrosine motif, [F/Y]XNPX[Y/F]. Kir1.1 is recruited into CCPs by phosphotyrosine-binding (PTB) domain-containing CLASPs, Disabled-2 (Dab2), and the autosomal recessive hypercholesterolemia (ARH) protein [24].

Furthermore, some di-leucine motifs [D/E]XXXL[L/I] are recognized by AP-2. The TWIK-related acid-sensitive K^+^ (TASK) channel 3.1 belongs to a family of two-pore domain K^+^ channels (K_2P_3.1). K_2P_3.1 contains two tyrosine-based motifs and one di-leucine (EHRAL_263_L) motif. Nerve growth factor (NGF) triggers the CME of K_2P_3.1, and in contrast to the tyrosine-based motifs, the di-leucine signal is partially responsible for the endocytosis [25]. 

#### 2.1.2. Ubiquitination

This reversible posttranslational modification (PTM) involves the covalent attachment of ubiquitin (UBQ), a small 8 kDa protein, to lysine residues of a target protein. UBQ is a highly conserved 76 amino acid protein expressed in all eukaryotic cells. Ubiquitination can regulate a wide variety of processes, from protein degradation by proteasome, DNA repair, and transcription, to membrane trafficking acting as an endocytic signal [26,27]. Ubiquitination involves the sequential action of three families of enzymes: E1 or ubiquitin-activating, E2 or ubiquitin-conjugating, and E3 or ubiquitin-ligase enzymes. UBQ can be removed by peptidases named deubiquitinating enzymes (DUBs) [28].

Proteins can be differentially ubiquitinated: (i) monoubiquitination (the attachment of one UBQ moiety to a single lysine residue), (ii) multi-monoubiquitination (the attachment of one UBQ to several lysine residues of a substrate), and (iii) polyubiquitination (the attachment of additional UBQ molecules to UBQ(s) on a lysine residue to form a UBQ chain) [26,27,28,29,30]. Although UBQ contains 7 lysine residues for self-ubiquitination, Lys^48^- and Lys^63^-linked chains are the most abundant. Polyubiquitinated Lys^48^-linked chains mainly play a role in proteasomal degradation. Polyubiquitinated Lys^63^-linked chains target a protein for lysosomal degradation, induce the endocytosis of membrane proteins, and are involved in DNA damage repair, ribosomal function, and NF-κB signaling [26,30,31]. The UBQ signal is recognized by specific domains called ubiquitin-binding domains (UBDs) or ubiquitin-interacting motifs (UIMs) [32]. Among the adaptors that contain UBDs, we focus on mammalian Epsin1, 2, and 3 and their binding partners Eps15 and Eps15R. In general, Epsins and Eps15/R are adaptors that are believed to cooperate in the recruitment of ubiquitinated cargo to CCPs. Moreover, Epsins are thought to contribute to membrane curvature during CCV formation [31,33].

The epithelial sodium channel (ENaC) is one of the most characterized ubiquitinated IChs. ENaC is a protein complex consisting of 4 subunits (2 α, β and γ subunits) expressed in the apical membrane of epithelial tissues with low luminal Na^+^ concentrations, such as the lungs, colon, and distal kidneys [34]. CME and ubiquitination contribute to ENaC internalization. The channel subunits interact with Epsin, and both ENaC and Epsin interact with clathrin adaptors. The overexpression of Epsin downregulates ENaC activity in CHO cells and Xenopus oocytes. This effect is dependent on the UBD of Epsin and ENaC ubiquitination and its interaction with the E3 ubiquitin ligase Nedd4-2 [35,36].

### 2.2. Clathrin-Independent Endocytosis

Clathrin-independent endocytosis (CIE) comprises the other internalization pathways that do not require the clathrin-associated machinery a clathrin coat. This mechanism was first described for the entry of bacterial toxins. However, a variety of cargo proteins can enter cells by CIE, such as transporters, IChs, cell adhesion molecules, and immune cell receptors [37].

CIE facilitates two types of endocytosis: large micrometer-scale pathways (macropinocytosis and phagocytosis) and a spectrum of smaller (<200 nm) scale processes [38]. The latter group can be further classified by whether dynamin, a large GTPase, participates—dynamin-dependent or dynamin-independent mechanisms – or whether other components of the endocytic machinery, such as small GTPases (RhoA, CDC42/ARF1 and ARF6-regulated), are involved [39]. In addition, evidence suggests that other, yet unidentified, CIE pathways might be involved.

#### 2.2.1. Mechanisms Dependent on Caveolin

Caveolae-mediated endocytosis is the best-characterized dynamin-dependent CIE pathway. Caveolae are specialized membrane invaginations (50–80 nm) marked by the presence of caveolin, an integral membrane protein (21 kDa) with cytosolic N- and C-terminal domains connected by a hydrophobic sequence. Caveolae, enriched in sphingolipids and cholesterol, concentrate signaling receptors and effectors [38,39].

The Kir6.1 channel, the major vascular K_ATP_ isoform, is mainly localized in the caveolae of aortic smooth muscle cells. Caveolae disruption with MβCD or caveolin-1 siRNA prevents the PKC-induced internalization of Kir6.1, suggesting that caveolae compartmentalization plays a functional role [40]. The transient receptor potential vanilloid 5 (TRPV5) channel is a gatekeeper for transepithelial Ca^2+^ reabsorption into the kidney. TRPV5 undergoes constitutive caveolin-dependent endocytosis, and PKC activation inhibits its caveolin-dependent internalization, leading to an increase in channel cell surface abundance [41].

The analysis of caveolar endocytosis is rather difficult because the same endocytic cargo may internalize by different mechanisms or may switch pathways under different conditions. In this context, the evidence indicates that TRPV5, which undergoes caveolin-dependent endocytosis, is also partially internalized by CME to enter a Ca^2+^-controlled recycling pathway [42]. In this complex scenario, the renal outer medullary Kir1.1 channel, critical for K^+^ secretion into cortical collecting ducts, is another example. As described above, the C-terminus of Kir1.1 contains the tyrosine-based NPXY internalization motif ([F/Y]XNPX[Y/F]) involved in CME. In addition, Kir1.1 interacts with clathrin and α-adaptin (an AP-2 subunit) [24,43]. However, evidence suggests a CIE alternative. Thus, caveolin-1 decreases Kir1.1 abundance at the plasma membrane and inhibits channel activity. The deletion of the Kir1.1 clathrin-endocytosis motif fails to abolish the effect of caveolin-1 on channel activity. Moreover, the expression of microRNA 802, which suppresses caveolin-1, increases channel activity [44].

#### 2.2.2. The RhoA-Dependent Mechanism

Another internalization mechanism is mediated by dynamin and dependent on the small GTPase RhoA. The RhoA-dependent mechanism was identified during the study of interleukin-2 receptor (IL-2R-β) internalization [39]. Kv1.2 is a member of the voltage-gated potassium channel, Shaker subfamily. RhoA suppresses Kv1.2 currents by modulating channel endocytosis. This regulation takes place through two different pathways: (i) clathrin-dependent and (ii) cholesterol-dependent mechanisms. The activation of Rho kinase (a RhoA effector, ROCK) via the lysophosphatidic acid (LPA) receptor triggers the CME of Kv1.2. This effect is blocked either by ROCK inhibition or clathrin RNA interference. However, constitutive Kv1.2 endocytosis is highly dependent on cholesterol levels and ROCK activity. The inhibition of ROCK and cholesterol alteration by filipin increases Kv1.2 membrane expression, whereas clathrin RNA interference shows no additive effect [45].

#### 2.2.3. The ARF6-Dependent Pathway

The ARF6-regulated mechanism is a dynamin-independent CIE pathway. Some IChs containing acidic clusters are recruited into the ARF6-regulated recycling pathway. The Kir3.4 channel, which has two types of acidic motifs (potassium acidic clusters, KACs), localizes into ARF6-positive vacuolar structures; however, a KAC-deleted mutant failed to enter an ARF6 compartment. Evidence suggests that cargo proteins entering an ARF6 structure can be either recycled to the cell surface or redirected to Rab5 endosomes. In this way, Kir3.4 inside an ARF6 compartment is recycled back to the plasma membrane rather than routed to Rab5 endosomes [46].

The hERG (human ether-a-go-go related gene) potassium channel (Kv11.1) is critical for the repolarization of the cardiac action potential. A reduction in Kv11.1 cell surface expression correlates with long QT syndrome and an increased risk of acquiring ventricular arrhythmias [47]. The channel undergoes rapid internalization into endosomes, but neither dynamin inhibition nor dominant negative Rab5 prevent its internalization. However, cholesterol depletion by MβCD and inhibition of ARF6 activity significantly affect endocytosis [48]. 

## 3. Stimulus-Induced Endocytosis of Ion Channels

### 3.1. Receptor-Mediated Internalization

Receptor activation triggers PTM of IChs, such as phosphorylation and ubiquitination, which induce internalization. Kv1.2 undergoes tyrosine phosphorylation and functional suppression through the activated M_1_ muscarinic ACh receptor (mAChR) and stimulated epidermal growth factor receptor (EGFR) [49]. The receptor-mediated tyrosine phosphorylation of Kv1.2 reduces its interaction with cortactin, which links the channel to the actin cytoskeleton, thereby leading to endocytosis and a decreased Kv1.2 current [50]. Similarly, EGFR activation leads to Kv1.3 phosphorylation [51], but in contrast to Kv1.2 phosphorylation, Kv1.3 endocytosis is independent of this tyrosine phosphorylation. EGFR signals induce the downregulation of Kv1.3 via two complementary mechanisms: (i) tyrosine phosphorylation of the channel reduces the Kv1.3 current, and (ii) an unconventional ERK1/2 kinase-dependent mechanism triggers channel endocytosis [52]. In contrast to their effect on Kv1.3 and Kv1.2, the activation of tyrosine kinase receptors enhances, rather than suppresses, the Kv7.1 current [53]. Kv7.1, in association with the KCNE1 β-subunit, recapitulates the cardiac I_Ks_ current. Stimulation by α_1_-AR suppresses the Kv7.1/KCNE1 current and triggers the ubiquitin-dependent CME by activating AMPK [14].

### 3.2. Drug-Induced Endocytosis

Kv11.1, which is critical for cardiac action potential repolarization, must be tested in preclinical safety assays for all potential drugs. Acquired long QT syndrome is the most common adverse cardiac effect caused by antidepressants. This pathology has been related to syncope, Torsade de Pointes arrhythmias, and sudden cardiac death. Long QT syndrome might be caused by a direct blockade of Kv11.1 or by indirect inhibition of its trafficking [9,54]. Desipramine, a tricyclic antidepressant, simultaneously blocks and indirectly inhibits the channel causing acquired long QT syndrome. Desipramine increases ubiquitin-dependent Kv11.1 internalization and degradation, and impedes channel trafficking from the endoplasmic reticulum [55].

Quinidine, a class I antiarrhythmic drug, triggers Kv1.5 endocytosis. Kv1.5, participating in ultrarapid cardiac K^+^ current (I_Kur_), controls the duration of the atrial action potential. The treatment of human atrial myocytes with quinidine triggers Kv1.5 internalization and simultaneously blocks the channel. Moreover, acute quinidine-induced endocytosis is reversible, whereas chronic treatment results in channel proteasomal degradation [56].

### 3.3. Low K^+^-Induced Endocytosis

A reduction in extracellular K^+^ concentration (hypokalemia) is a risk factor for long QT syndrome. Thus, low extracellular [K^+^] triggers Kv11.1 channel degradation through the multivesicular body (MVB)/lysosomal pathway. Upon the reduction of extracellular [K^+^], Kv11.1 undergoes a conformational change, which results in channel degradation. Kv11.1 endocytosis is concomitant with the monoubiquitination of the channel and is dependent on caveolin [57]. However, as explained above, the study of caveolar endocytosis can be complex because of overlapping alternative mechanisms. Thus, the ARF6-mediated mechanism may also be involved in the Kv11.1 recycling pathway [48]. In fact, Kv11.1 channels coimmunoprecipitate and colocalize with caveolin-1 (Cav1) under hypokalemic conditions. Moreover, the knockdown of Cav1 hampers the endocytosis of Kv11.1 channels in HEK-293 cells. Furthermore, knocking down caveolin-3 (Cav3) has been shown to prevent the low K^+^-induced reduction in the Kv11.1 current in cultured neonatal rat ventricular myocytes [58].

## 4. The Components of Ion Channel Endocytosis

### 4.1. Ubiquitin Ligases and Deubiquinating Enzymes

The UBQ system regulates many cellular processes and is of major interest because of the ubiquitin-mediated degradation of IChs. E3 ubiquitin ligases are crucial to target selection for ubiquitination. Ubiquitin ligases are structurally divided into 4 groups. The two main groups are (i) HECT-type E3 ligases, which contain a HECT (homologous to E6-AP COOH terminus) domain that forms a thioester bond with UBQ to ubiquitinate the target protein directly, and (ii) RING finger E3 ligases, which contain a RING (really interesting new gene) finger domain, acting as adaptor proteins for the E2 ligases and facilitating the transfer of UBQ from E2 directly to the substrate [29,59,60].

The Nedd4/Nedd4-like protein family consists of the major ICh negative regulators. ENaC [61], CLC-2 [62], some Na_v_ (voltage-gated sodium channels) [63], Kv1.3 [64], Kv7.1 [65], Kv7.2/Kv7.3 [66], and Kv11.1 [67] are regulated by Nedd4-2. The Nedd4/Nedd4-like family comprises 9 members in humans and belongs to the HECT-type ubiquitin ligase group [59,68]. An altered Nedd4-2 function is implicated in hypertensive disorders such as Liddle’s syndrome [69], epilepsy [70], neuropathic pain, and long QT syndrome [71]. The Nedd4-like family exhibits a catalytic HECT domain at the C-terminus; a C2 domain at the N-terminus, which binds to phospholipids in a calcium-dependent manner; and from 2 to 4 tandem WW (tryptophan) domains, which mediate binding to specific substrates. Two WW domains specifically interact with conserved PY motifs (PPXY or LPXY sequences) or proline-rich regions in proteins [72]. ENaC is clearly the best-studied example of a ubiquitinated ICh by the Nedd4-like family. The C-terminal regions of each channel subunit contain a PY motif (PPXYXXΦ), which overlaps with a tyrosine-based motif (YXXΦ) related to CME. The mutation of the ENaC PY motifs causes Liddle’s syndrome, an inherited hypertension disorder that is triggered by increased channel activity. The PY motif of ENaC recruits Nedd4-2 and downregulates channel surface expression via ubiquitination. In Liddle’s disease, the loss of the PY-binding motif where Nedd4-2 attaches reduces the ubiquitination of ENaC and, consequently, increases the number of channels at the plasma membrane [73,74]. More interestingly, the serum and glucocorticoid-regulated kinase (SGK), a downstream mediator of aldosterone, reduces the binding of Nedd4-2 to ENaC by phosphorylating Nedd4-2 and thus also increases ENaC cell surface expression and regulates epithelial Na^+^ absorption [75].

Nedd4-2 also targets Na_v_ channels, which are critical for maintaining the action potential in most excitable cells. Among the 10 isoforms of Na_v_s channels, 7 contain a PY motif in the C-terminus [63]. Laedermann and colleagues, by using the spread nerve injury (SNI) model of traumatic nerve injury-induced neuropathic pain, showed a reduction in Nedd4-2 expression in dorsal root ganglion (DRG) cells associated with an increase in Na_v_1.7 and Na_v_1.8 expression and function. In addition, the nociceptive DRG neuron-specific knockout of Nedd4-2 triggered the dysregulation of Na_v_1.7 and Na_v_1.8 expression, similar to that obtained with the SNI model. Moreover, animals show an altered nociceptive pain phenotype, which is a feature of peripheral neuropathic pain, characterized by the dysregulation of Na_v_ channels [76].

Nevertheless, the presence of a PY motif is not always obligatory for Nedd4-like function. For example, Kv1.3 [13,64,77] and Kv1.5 [78] have no canonical PY motifs. This evidence suggests either alternative mechanisms of binding or the presence of yet undiscovered intermediate adaptors that facilitate the interaction of a ubiquitin ligase and a target.

In addition to ubiquitin ligases acting at the plasma membrane, other E3 enzymes can be recruited into the early endosomes (EEs) and ubiquitinate cargo proteins prior to sorting. The sorting in the EEs is mediated by the endosomal sorting complex required for transport (ESCRTs). ESCRT-0 contacts the ubiquitinated cargo, and ubiquitination is necessary for sorting. Whether the cargo is ubiquitinated before EE formation the target can be degraded [79]. c-Cbl is a member of the RING finger E3 ligase family with an N-terminal tyrosine kinase-binding domain (TKB) and an extended C-terminal tail with proline-rich motifs and a UBD. c-Cbl regulates CFTR by two mechanisms: (i) acting as an adaptor protein facilitating CFTR endocytosis by a UBQ-independent mechanism, and (ii) ubiquitinating CFTR in EEs to promote lysosomal degradation [80].

Moreover, there is dynamic interplay among ubiquitinating and DUB enzymes, which fine-tune the ubiquitin cascade by cleaving UBQ from substrates and editing UBQ chains, processing ubiquitin precursors and inhibiting E2 and E3 enzymes. Proteasome-related DUBs contribute to the prevention of ubiquitinated protein degradation, whereas lysosome-associated DUBs are crucial in receptor degradation and recycling. DUBs are classified according to their sequences and domain conservation in 6 families, with the largest family consisting of ubiquitin-specific proteases (USPs) [81]. As explained above, ENaC is ubiquitinated by Nedd4 family members, a process that can be reversed by USP2-45. USP2-45, induced by aldosterone, deubiquitinates ENaC and increases channel abundance [82]. USP2-42 also interacts with ENaC to moderate increases in channel surface expression and activity [83]. USP2 isoforms also regulate Kv7.1. Krzystanek and colleagues described the antagonism between USP2-45 and USP2-63 on Nedd4-2-dependent Kv7.1 ubiquitination, showing the restoration of the channel localization at the plasma membrane. Evidence suggests that the balance between the ubiquitination/deubiquitination of Kv7.1 ultimately controls the duration of the cardiac action potential and the potassium flux across the membrane of epithelial cells [84]. Finally, USP10, located in EEs, regulates the deubiquitination of CFTR, facilitating the recycling of the channel to the plasma membrane. USP10 decreases the ubiquitination of CFTR, whereas the knockdown of USP10 promotes the ubiquitination and reduction of CFTR at the cell surface [85].

### 4.2. β-Arrestins

β-Arrestin 1 and 2, clathrin adaptors that recognize cargo protein, are ubiquitously expressed in mammalian cells. β-Arrestins desensitize members of the G-protein coupled receptor (GPCR) family [86,87]. In addition, β-arrestins act as multifunctional adaptors mediating trafficking and signal transduction, not only through GPCRs but also through other receptors and IChs [88]. β-Arrestins, through interactions with clathrin and AP-2, act as adaptors for the agonist-induced endocytosis of many GPCRs, promoting the accumulation of cargo protein in CCVs [89]. β-Arrestins contain clathrin and AP-2 binding motifs and a domain capable of cargo (GPCR) binding. Interestingly, upon binding to GPCRs and membranes, β-arrestins undergo conformational changes facilitating their interactions with other signaling and trafficking proteins. In addition to conformational changes, β-arrestins undergo PTM, such as phosphorylation and ubiquitination [88].

The inward rectifying potassium channel 3 (Kir3) controls neuronal excitability in response to GPCR activation. Kir3.1/3.2 subunits form signaling complexes with the delta opioid receptor (DOR). Upon receptor stimulation, the complex provides a platform for β-arrestin 2 association with receptors and channels, which drives their internalization by CME [90]. Similarly, sustained angiotensin II stimulation of angiotensin receptor type 1 (AT1R) on the Ca_v_1.2 calcium channel induces β-arrestin 1 recruitment to the channel complex, causing the internalization of Ca_v_1.2 in T-tubules. Therefore, L-type calcium currents decrease by ≈60%, and calcium transient amplitude and action potential duration decrease [91].

Moreover, β-arrestins act as adaptors for ubiquitin ligases mediating the ubiquitination and degradation of targets [88]. TRPV4 is a member of the vanilloid subfamily of the transient receptor potential family, which is present in cardiovascular tissues and epithelial cells. TRPV4-mediated entry of Ca^2+^ into endothelial cells seems to be important for nitric oxide production, vasoconstriction, and vasodilatation of peripheral blood vessels. AT1R forms a complex with TRPV4 in vascular smooth muscle cells. Upon angiotensin stimulation, TRPV4 is ubiquitinated via β-arrestin 1, which interacts with the E3 ligase AIP4, leading to channel internalization. The annihilation of β-arrestin 1 impairs ubiquitination and angiotensin-induced TRPV4 internalization [92]. The presence of β-arrestin 1 is also essential for Na^+^/H^+^ exchanger 1 (NHE1) ubiquitination by Nedd4-1. siRNAs against either Nedd4-1 or β-arrestin 1 reduce NHE1 ubiquitination and endocytosis, resulting in an increase in NHE1 plasma membrane abundance. Because NHE1 lacks the PY motif and β-arrestins are scaffolding proteins for ubiquitin ligases, it is likely that β-arrestin 1 recruits Nedd4-1 to the C-terminus of NHE1 for subsequent ubiquitination [93].

### 4.3. Rab Proteins

Rab proteins are monomeric small GTPases involved in the regulation of vesicular transport in all eukaryotic cells, including vesicle formation and trafficking between different intracellular compartments, docking vesicles to their target membrane, and recycling proteins back to the cell surface. To date, more than 60 Rabs have been identified in mammalian cells, which reflects the complexity of these transport pathways [94,95]. IChs, once located in endosomes, can be either degraded or recycled back to the membrane. In this scenario, Rabs participate in the regulation of the trafficking pathways and consequently modulate the expression of IChs at the plasma membrane.

#### 4.3.1. Rab4

Once a membrane protein internalizes into EEs, it can be recycled back to the cell surface. This mechanism might rely on tubule–vesicular transport carriers, known as fast recycling, or by transit via the endocytic recycling compartment, known as slow recycling [10]. The fast pathway for recycling depends on Rab4-GTP [96]. Rab5 and Rab4 act together to regulate cargo entry to and exit from EEs, respectively [94]. Thus, the overexpression of Rab4 increases Kv1.5 current in myoblasts, suggesting that Rab4 participates in the recycling of Kv1.5 back to the plasma membrane after internalization [97]. Rab4 also regulates the recycling of the Transient Receptor Potential Canonical 1 (TRPC1) via the fast recycling pathway. The overexpression of Rab4 rescues TRPC1 from EE and routes the channel to ER-plasma membrane junctions [98]. In contrast to its action on Kv1.5, Rab4 significantly decreases the expression of Kv11.1 at the plasma membrane due to an increase in Nedd4-2 expression [99]. On the other hand, high levels of Rab4 also reduce the plasma membrane abundance of ENaC and CFTR in epithelial cells, but the molecular mechanisms of these functions are as of yet unknown [100].

#### 4.3.2. Rab5

Rab5 is important for the fusion of endocytic vesicles with EEs and homotypic EE fusion [101,102]. The early trafficking and internalization of Kv1.5 is dependent on Rab5. Internalized Kv1.5 colocalizes rapidly with Rab5, and the presence of dominant negative Rab5 (Rab5DN) blocks channel endocytosis and increases Kv1.5 current [97]. The overexpression of Rab5 or a constitutively active Rab5 mutant elevates TRPC1 retention in EE, suggesting that TRPC1 recycles by Rab5-dependent EE [98]. Rab5 also participates in the internalization of Kv7.1. Rab5 (N133I), a mutant with reduced GTP-binding affinity, increases I_Ks_ current upon stimulation of vesicle trafficking by the injection of GTP into Xenopus oocytes [103]. Moreover, Rab5 GTPase-mediated endosomal pathways regulate the transport of CFTR channels. Thus, Rab5aDN inhibits the internalization of CFTR and increases its cell surface abundance. The CFTR (ΔF508) channel is critical to approximately 90% of cystic fibrosis cases in Caucasian patients. Rab5aDN blocks endocytosis and enhances the accumulation of ΔF508 CFTR at the plasma membrane [100].

#### 4.3.3. Rab7

Target protein routing for degradation continues through the endocytic pathway during the maturation of EEs into late endosomes (LEs), which ultimately fuse with lysosomes [10]. Rab7 resides in late endosomes [104]. Kv1.5 colocalizes with Rab7, and a dominant negative of Rab7 (Rab7DN) increases the Kv1.5 current. Rab7 reduces Kv1.5 expression and activity in myoblasts, suggesting that a fraction of the internalized Kv1.5 is targeted for degradation [97]. Furthermore, the overexpression of Rab7 decreases the amount of intracellular and plasma membrane CFTR, whereas Rab7DN increases the intracellular pool of this channel. This evidence suggests that Rab7 promotes the transport of CFTR channels from EEs to LEs and, finally, to lysosomes, favoring their degradation [100].

#### 4.3.4. Rab11

In contrast to the fast recycling pathway, the slow recycling of channels to the plasma membrane is Rab11-dependent. Recycling endosomes (REs) cluster in the perinuclear-localized endocytic recycling compartment (ERC). REs often have a tubular shape and form a complex network of tubulo-vesicular endosomes [11]. REs are enriched in Rab11, which is also present in the trans-Golgi network and post-Golgi vesicles, where it is involved in trafficking proteins from juxta-Golgi endosomal compartments to the cell surface [105,106]. A fraction of the internalized Kv1.5 colocalizes with Rab11-positive endosomes but, in contrast to Rab4, Rab11 is involved in prolonged periods of internalization [97]. Kv11.1 is also recycled via the Rab11 pathway. The low K^+^-mediated reduction of Kv11.1 is concomitant with an increased rate of channel recovery at the plasma membrane. This recovery reflects Rab11-dependent recycling rather than enhanced channel synthesis [107]. Rab11 also participates in the recycling of Kv7.1. SGK activation enhances the Rab11-mediated recycling of the Kv7.1/KCNE1 complex, increasing its membrane abundance [103]. ENaC also colocalizes with Rab11-associated recycling endosomes, which, in turn, increases channel activity by facilitating their recycling to the cell surface [108]. Rab11 is also involved in the endocytic recycling of CFTR channels. The overexpression of Rab11 moves both internalized CFTR and CFTR (ΔF508) to the plasma membrane [100]. Interestingly, evidence suggests that the Rab11-mediated mechanism involves the direct interaction of Rab11 with targets, such as TRPV5 [109].

## 5. Concluding Remarks

Endocytosis is crucial for the regulation of cell signaling and membrane dynamics (Table 1). The activity of IChs is strongly dependent on their abundance and proper surface location. A disruption of the balance between the secretory pathway and the endosomal network is associated with several diseases, such as Liddle’s and long QT syndromes, neonatal diabetes, and neuropathic pain. Clathrin-mediated endocytosis (CME) is the most characterized mechanism to date. Increasing evidence has led to an understanding of endocytic vesicle formation, membrane-bending mechanisms, and vesicle scission and release. Different internalization signals, playing roles in recruitment, have been identified for IChs functions. Channels exhibit tyrosine-based and dileucine linear motifs and undergo ubiquitination, which are recognized by different adaptors that lead to channel internalization.

Clathrin-independent endocytosis (CIE) is also a common mode of ICh internalization. However, the variety of mechanisms indicates a complex scenario. Many IChs are located in lipid rafts and caveolae, and their altered spatial distribution is related to human pathologies. In this context, caveolar-dependent internalization pathways, tightly connected with ICh function, are essential.

Specific insults trigger the massive internalization of IChs. The activation of receptors leads to the PTM of IChs and the recruitment of different mediators, such as β-arrestins and ubiquitin ligases. Moreover, hypokalemic conditions and some drugs can trigger the internalization of IChs. The ubiquitin-mediated endocytosis-linked degradation of ICh deserves considerable attention because of the essential role of ubiquitin biology in diseases, such as neurodegenerative disorders, in which vital ICh functions are dysregulated. The dynamic interplay between ubiquitin ligases and DUB enzymes fine-tunes the ubiquitin system with high substrate specificity. The Nedd4-like family consists of the major IChs negative regulators, but not all targets have a PY binding motif. Therefore, alternative binding mechanisms and intermediate adaptors deserve further research.

**Figure 2:**
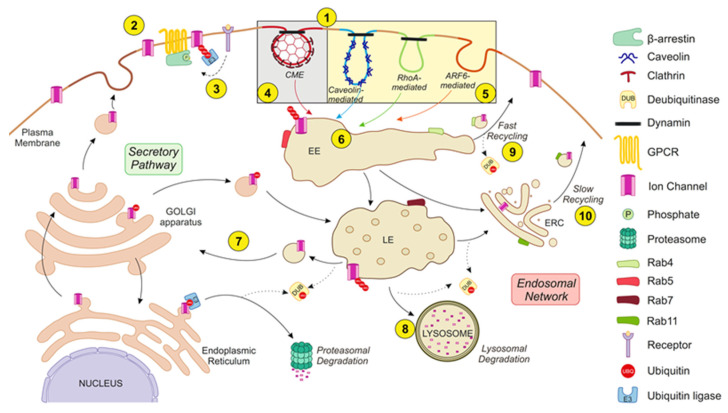
The endocytic ICh network. The balance between the secretory pathway and the endosomal network regulates ICh abundance at the plasma membrane. The endocytic network starts with the internalization of protein cargo by distinct mechanisms (**1**). ICh exhibits constitutive turnover but undergoes massive internalization upon specific stimulation. Thus, receptor activation (e.g., GPCR activation) provides a platform by which β-arrestins associate with receptors and ICh to mediate channel endocytosis (**2**). Receptor stimulation also triggers posttranslational modifications of IChs, such as phosphorylation (P) and ubiquitination (UBQ), which is realized through specific kinases and ubiquitin ligases (E3), respectively, which induce ICh endocytosis (**3**). Different internalization routes for IChs can be classified by their dependence on clathrin in (i) clathrin-mediated endocytosis (CME) (**4**) or (ii) clathrin-independent endocytosis (CIE) (**5**). CME is the most common internalization pathway for IChs. CIE mechanisms can be classified as caveolin-, RhoA- or ARF6-mediated. Internalized vesicles fuse to early endosomes (EEs) and are partially sorted (**6**). Target channels can be recycled (either back to the plasma membrane or to the secretory pathway) (**7**) or degraded in lysosomes (**8**). Recycling to the plasma membrane is achieved through tubule-vesicular transport carriers, known as the “fast recycling” process (**9**), or by the endocytic recycling compartment (ERC), known as the “slow recycling” process (**10**). Moreover, vesicles can bypass the trans-Golgi network and enter the secretory pathway (**7**). Therefore, there is an interplay between E3 ligases and deubiquitinating (DUB) enzymes. Ubiquitin is released from ubiquitinated substrates by DUBs. IChs sorted for degradation continue to move through the endocytic pathway from the EEs to the late endosomes (LEs) and are ultimately degraded by lysosomes. Rab proteins guide these turnover stages. Rab5 and Rab4 collaborate to regulate channel entry into and exit from EEs, respectively. Thus, Rab5 regulates the fusion of endocytic vesicles with EEs, whereas Rab4 is involved in the “fast recycling” pathway. On the other hand, Rab7 mediates the vacuolar fusion of EEs with LEs, and Rab11 participates in the “slow recycling” pathway. See the text for further details.

Nevertheless, internalization might not be the final fate of IChs. Targeted channels, once in endosomes, can be sorted for degradation or recycled back to the membrane. Rab proteins are involved in several steps of this turnover mechanism (Figure 2).

ICh turnover is a complex process triggered by a variety of stimuli that require the coordinated action of a large variety of proteins. Endocytosis fine-tunes the plasma membrane abundance of IChs, which is key in health and disease. Aberrant ICh turnover is associated with human disorders and is especially linked with heart and brain maladies such as long QT syndrome and neuropathic pain, respectively. Moreover, a reduced insulin secretion in permanent neonatal diabetes patients affects glucose metabolism and severe hypertension is observed in Liddle’s syndrome patients. Since IChs are expressed in all cell types, alterations in their endocytic traffic surely affect a wide range of functions not yet understood. Therefore, gaining knowledge about the different mechanisms and players involved in ICh turnover is worth the effort.

## Figures and Tables

**Table 1:** Ion channels and their endocytic pathways.

Ion Channel	Type of Endocytosis	Stimulus of Internalization	Ubiquitination	Rabs	Associated Pathologies
ENaC	CME [12]	Kidney: Basal conditions (low aldosterone) [75] Alveoli: hypercapnia [110]	Polyubiquitination [74] E3 Nedd4-2 [75] DUB USP2-45 [82,83]	Rab4 [111] Rab11 [108]	Liddle’s syndrome [69]
Na_v_ family (Na_v_1.1-1.3 and 1.5-1.8)	ND	ND	Polyubiquitination E3 Nedd4-2 [63]	ND	Congenital type 3 long QT syndrome # (Na_v_1.5) [71] Neuropathic pain (Na_v_1.7-1.8) [76]
Kv1.2	CME, RhoA and cholesterol-dependent [45]	Constitutive [45] and Receptor-mediated: mAChR [112] and EGFR [113]	ND	ND	ND
Kv1.3	CME [13,52]	Receptor-mediated (EGFR) [52] and PKC [13]	Polyubiquitination E3 Nedd4-2 * [13,64,77]	ND	ND
Kv1.5	CME ‡ [97] Caveolin-mediated [114]	Receptor-mediated (5-HT) [114], Drug-induced (quinidine) [56], PKC and AMPK [115]	Ubiquitination E3 Nedd4-2 * [78]	Rab4, Rab5, Rab7, Rab11 [97]	ND
Kv7.1	CME [14]	Receptor-mediated (α_1_AR) [14] and PKC [116]	Polyubiquitination E3 Nedd4-2 [14,65] DUB USP2-45 and USP2-63 [84]	Rab5, Rab11 [103]	Type 1 long QT syndrome [117]
Kv7.2/7.3	CME [118]	Receptor and calpain-mediated (NMDA—high glutamate) [118]	Polyubiquitination E3 Nedd4-2 [66] DUB USP36 [119]	ND	ND
Kv11.1	Caveolin [57,58] ARF6-mediated [48]	Drug (Desipramine) [55] and Low K^+^-induced [57]	Monoubiquitination E3 Nedd4-2 [67]	Rab4 [99] Rab11 [107]	Acquired long QT syndrome [55]
Kir1.1 (ROMK)	CME [24] Caveolin-mediated [44]	Constitutive [24], WNK [24] and PTK kinases [120]	Monoubiquitination [120] E3 Nedd4-2 * [121] CIE: E3 POSH [122]	ND	ND
Kir2.3	CME [17]	Constitutive [17]	ND	Rab11 [123]	ND
Kir3.1/3.2	CME [90]	Receptor-mediated (DOR) [90]	ND	Rab7 [124]	ND
Kir3.4	ARF6-mediated [46]	ND	ND	Rab7 [124]	ND
Kir6.1	Caveolin-mediated [40]	PKC [40]	ND	ND	ND
Kir6.2	CME [7]	PKC [125]	Ubiquitination [126]	Rab7 [125]	Permanent neonatal diabetes mellitus [7]
CFTR	CME [15]	ND	Polyubiqutination E3 CHIP [127], c-Cbl [80] and Nedd4-2 * (ΔF508) [128] DUB USP10 [85]	Rab4, Rab5, Rab7, Rab11 [100]	ND
CLC-2	Dynamin-dependent [129]	ND	Polyubiquitination E3 Nedd4-2 * [62]	Rab5, Rab11 [129]	ND
TRPC1	ARF6-mediated [98]	ND	ND	Rab4, Rab5 [98]	ND
TRPV4	CME ‡ [92]	Receptor-mediated (AT1R) [92]	Ubiquitination E3 AIP4 [92]	ND	ND
TRPV5	CME [42] Caveolin-mediated [41]	Constitutive [41]	E3 Nedd4-2 * [130]	Rab11 [42]	ND
Ca_v_1.2	CME [16]	Receptor-mediated: AT1R [91] and ERα [131]	Polyubiquitination E3 Mdm2 [131]	Rab11 [16]	ND

ND: not determined. * Channels without PY motif. # Congenital type 3 long QT syndrome: Evidence in heterologous system but not in animals. ‡ Indirect evidence.
